# Antiproliferative, Antiangiogenic, and Antimigrastatic Effects of *Oroxylum indicum* (L.) Kurz Extract on Breast Cancer Cell

**DOI:** 10.1155/2023/6602524

**Published:** 2023-07-08

**Authors:** Benjamas Chiraatthakit, Benjawan Dunkunthod, Sanong Suksaweang, Griangsak Eumkeb

**Affiliations:** ^1^School of Preclinical Sciences, Institute of Science, Suranaree University of Technology, Nakhon Ratchasima 30000, Thailand; ^2^Thai Traditional Medicine Program, Faculty of Nursing and Allied Health Sciences, Phetchaburi Rajabhat University, Phetchaburi 76000, Thailand; ^3^Department of Pathology and Laboratory Medicine, Institute of Medicine, Suranaree University of Technology, Nakhon Ratchasima 30000, Thailand

## Abstract

Breast cancer recurrence continues to pose a major clinical problem, despite significant advancements in early diagnosis and an aggressive mode of treatment. This study aimed at investigating the anticancer activity of *Oroxylum indicum* extract (OIE) by assessing cell proliferation, cell migration, and angiogenesis in metastatic breast cancer MDA-MB-231 cell lines. This study also estimated the phytochemical profiles of OIE by LC-QTOF-MS. The extract was found to contain six identified flavonoid substances, and baicalein was the most abundant substance in the extract. Cell proliferation capacity was performed by cell counting kit-8 (CCK-8) and colony formation assays. The effect of OIE on cell migration was determined using wound healing and transwell assays. Meanwhile, MDA-MB-231-induced angiogenesis on chick chorioallantoic membrane (CAM) was applied to investigate the *ex vivo* antiangiogenesis activity of the extracts. OIE at concentrations lower than 600 *μ*g/mL had no cytotoxic effects against MDA-MB-231 cells. OIE was found to inhibit the long-term colony formation ability of MDA-MB-231 cells in a concentration-dependent manner. Antimigration and antiangiogenesis activities were further investigated using noncytotoxic concentrations of OIE ranging from 25 to 150 *μ*g/mL. OIE greatly reduced the migration of MDA-MB-231 breast cancer cells in a dose-dependent manner. OIE significantly suppressed the MDA-MB-231-induced angiogenesis, and there was no substantial toxic effect on natural angiogenesis. Interestingly, the concentration of OIE at 150 *μ*g/mL was as practically potent as pazopanib, the positive anticancer drug, at 4.37 *μ*g/mL in inhibiting MDA-MB-231 cell migration and angiogenesis induced by these cells. Therefore, the inhibitory effects of OIE in cell proliferation and cell migration, together with antiangiogenesis in MDA-MB-231 breast cancer cells, suggesting that OIE has the potential to be a novel adjunct candidate for breast cancer chemotherapeutic agents.

## 1. Introduction

Breast cancer is the leading cause of cancer-associated mortality among women [1]. Breast cancer treatment can become complicated if the tumor cells metastasize to distant body organs [1, 2]. According to Hanahan and Weinberg, inducing angiogenesis and the ability to invade surrounding tissues and metastasize are hallmarks of tumor malignancy [3]. Typically, cancer cells secrete various proangiogenic factors such as vascular endothelial growth factors (VEGFs), fibroblast growth factor-2 (FGF-2), and transforming growth factor-alpha (TGF-*α*), which can activate proangiogenic signalling pathways to promote tumor growth, invasion, metastasis, and angiogenesis [4]. An increased level of angiogenesis is associated with decreased survival in breast cancer patients [5]. If angiogenesis is a critical and rate-limiting step in tumor progression, then blocking angiogenesis should inhibit cancer progression. It is, therefore, essential to develop new agents with high efficacy to block cancer angiogenesis and metastasis processes explicitly.

*Oroxylum indicum* (L.) Kurz (*O. indicum*) has been used in the Indian Ayurvedic medicine system to cure stomach problems, diarrhea, dysentery, and rheumatism [6, 7]. Numerous studies conducted on phytochemical investigations of *O. indicum* clearly demonstrated the abundance of natural flavonoids such as baicalein, chrysin, and oroxylin A [6, 8, 9]. *O. indicum* and its flavonoid constituents have been reported to possess a wide range of biological and pharmacological activities [8, 10–15]. Over recent years, *O. indicum* has attracted a growing interest in treating various cancer cells, focusing on inhibiting cell division and proliferation [6, 16–20]. Talari et al. have demonstrated the antiangiogenic effect of *O. indicum* on VEGF-induced neovascularization in the CAM model [21]. However, aggressive breast cancer cells may also express many proangiogenic factors which induce cancer cells to form new blood vessels as a route for metastasis [4]. Therefore, we first investigated the antiangiogenesis effect of OIE on aggressive MDA-MB-231-induced neovascularization in the CAM model and assessed the effect of OIE on cell proliferation, colony formation, and migration of these cells.

## 2. Materials and Methods

### 2.1. Cell Lines

The human breast cancer MDA-MB-231 cell line was purchased from the American Type Culture Collection (ATCC). The cells were grown in L-15 supplemented with 10% heat-inactivated fetal bovine serum (FBS), 100 IU/mL penicillin, 100 mg/mL streptomycin, and 2 mM L-glutamine. Cultures were maintained in a humidified atmosphere with 5% CO_2_ at 37°C.

### 2.2. Preparation of *O. indicum* Extract (OIE)

*O. indicum* (fruit pods) fresh samples were purchased from the local market in Wang Nam Khiao District, Nakhon Ratchasima Province, Thailand. The voucher specimens were kept in the flora of the Suranaree University of Technology Herbarium (SOI0808U). The plant extraction was conducted according to a previous study [22]. The *O. indicum* extract (OIE) was stored at −20°C until use in subsequent experiments.

### 2.3. Liquid Chromatography Coupled to Quadrupole Time-of-Flight Mass Spectrometry (LC-QTOF-MS) Analysis

The phytochemical profiling of OIE was analyzed using LC-QTOF-MS/MS in negative mode. The analyses were performed on the Dionex Ultimate 3000 UHPLC system (Dionex, USA) coupled with an electrospray ionization (ESI) tandem mass spectrometer (micrOTOF-Q II) (Bruker, Germany). A sample injection volume of 5 *μ*L was used for chromatographic separation of analytes using a Zorbax SB-C18 (250 mm × 4.6 mm × 3.5 *μ*m (Agilent Technologies, USA)) and a gradient program including deionized water containing 0.1% formic acid (FA) as solvent A and acetonitrile containing 0.1% formic acid (FA) as solvent B. The flow rate of the mobile phase was fixed at 0.8 mL/min and the temperature of the column was fixed at 35°C. The gradient program was optimized by passing through the reservoir 30% B, reaching 80% B at 30 min, and holding until 38 min, reducing at 30% B in 2 min, and holding until the run ended at 45 min. The LC-QTOF data were collected and processed by Compass 1.3 software (Bruker, Germany). Apiin, luteolin, quercetin, apigenin, kaempferol, baicalein, and oroxylin A were used as standard reference compounds. The calibration curves were constructed from peak areas of different concentrations of the reference standard (from 1 *μ*g/mL to 100 *μ*g/mL), and the concentrations of targeted compounds were calculated based on the equation for linear regression obtained from the calibration curves.

### 2.4. Cytotoxic Test (CCK-8 Assay)

The cell counting kit-8 (CCK-8, Cat. No. E-CK-A362, Elabscience Biotechnology Inc.) was performed to determine the cell viability of MDA-MB-231 cells after being treated with OIE, as previously described by Paquette et al. [23]. Briefly, MDA-MB-231 cells (1.5 × 10^4^ cells/well) were seeded in a 96-well plate for 24 hrs and treated with various concentrations of OIE. After 24 hrs, 10 *μ*L of CCK-8 solution was added to the cells and further incubated for 2 hrs at 37°C. The absorbance was measured at 450 nm using a microplate reader (Bio-Rad, USA).

### 2.5. Colony Formation Assay

The effect of OIE on the ability of MDA-MB-231 cells to form colonies was investigated using a colony formation assay as previously described by Brix et al. [24]. MDA-MB-231 cells (500 cells/well) were seeded for 24 hrs. Then, the cells were treated with the indicated concentration of OIE (50, 150, 300, and 600 *μ*g/mL), baicalein 3.75 *μ*g/mL, or pazopanib 4.37 *μ*g/mL for 24 hrs. After incubation, the culture medium was removed and washed twice with PBS. The culture medium was replaced every 2 days for two weeks. Then, the cells were washed twice with PBS, fixed with methanol: acetone (3 : 1), and stained with a 0.5% crystal violet solution for 15 min at room temperature. The number of colonies was photographed and counted.

### 2.6. Cell Migration Assay

#### 2.6.1. Wound Healing Assay

The effect of OIE on directional cell migration *in vitro* was evaluated by the wound healing assay [25]. Briefly, MDA-MB-231 cells were seeded at a density of 1 × 10^5^ cells/well in a 6-well plate and grown for 24 hrs. Next, a linear scratch wound was created across the middle of the well's surface using a 200 *μ*L pipette tip. After that, the culture medium was replaced with a fresh culture medium containing OIE (50, 150, 300, and 600 *μ*g/mL), baicalein 3.75 *μ*g/mL, or pazopanib 4.37 *μ*g/mL, and further incubated for 24 hrs. At predetermined time points (18 and 24 hrs), the images of cells that migrated into the denuded areas were taken using an inverted microscope with 40× magnification equipped with a DinoEye digital eyepiece. The widths of the wounds were measured using ImageJ software.

#### 2.6.2. Transwell Migration Assay

The effect of OIE on the migration ability of MDA-MB-231 cells and the *in vitro* migration assay were performed using the Transwell chamber system (6.5 mm diameter, 0.4 *μ*m pore size). Briefly, MDA-MB-231 cells (1 × 10^4^ cells/well) were seeded on the upper well of chambers placed in a 24-well plate in 450 *μ*L of serum-free medium containing indicated concentrations of OIE (25, 50, 100, and 150 *μ*g/mL), baicalein 3.75 *μ*g/mL or pazopanib 4.37 *μ*g/mL. The lower chamber was filled with 750 *μ*L of culture medium supplemented with 10% FBS. Cells were incubated undisturbed in the CO_2_ incubator for 18 hrs. The inserts' cells on the upper side were removed with a cotton swab. The migrated cells on the lower surface were fixed and stained with crystal violet. The images were captured, and the numbers of migrated cells were counted in four different microscopic fields. Results are expressed as the percentage of migrated cells compared to the control.

### 2.7. Chick Chorioallantoic Membrane (CAM) Assay

#### 2.7.1. The Toxicity of OIE on Natural Angiogenesis in the Chick Chorioallantoic Membrane (CAM) Model

The CAM assay was performed to evaluate the effect of OIE on natural angiogenesis at 24 and 48 hrs postexposure. Briefly, the fertilized chicken eggs were used after incubating them for 4 days. A 2 cm^2^ window was made in the shell to create a pocket to expose the CAM. A filter disc in the presence or absence of OIE with indicated concentrations (25, 50, 100, and 150 *μ*g/mL), baicalein 3.75 *μ*g/mL or pazopanib 4.37 *μ*g/mL was placed upon the CAM, and 20 *μ*L of 100 U/mL penicillin was immediately added. The exposed hole in each egg was closed with tape and further incubated for 24 and 48 hrs. After incubation, the CAM images were taken using a stereomicroscope with 40× magnification equipped with a DinoEye digital eyepiece and photographed. Angiogenesis was quantitated by counting the number of blood vessels in direct contact with the filter disk.

#### 2.7.2. The Antiangiogenic Effect of OIE on MDA-MB-231-Induced Angiogenesis in the CAM Model

The inhibitory effect of OIE on MDA-MB-231-induced angiogenesis was evaluated in the CAM model. Shortly, MDA-MB-231 cells at a density of 1 × 10^6^ cells were placed directly onto the CAM of the fertilized chicken eggs. Then, a filter disc in the presence or absence of OIE (25, 50, 100, and 150 *μ*g/mL), baicalein 3.75 *μ*g/mL, or pazopanib 4.37 *μ*g/mL, was placed upon the CAM before adding 20 *μ*L of 100 U/mL penicillin. The window was sealed with tape and incubated for 24 and 48 hrs. Angiogenesis at each time point was quantified.

### 2.8. Statistical Analysis

All statistical significance (Statistical Package for the Social Sciences, version 19) was determined by performing a one-way analysis of variance (ANOVA) with a Tukey's post hoc analysis to determine differences among each treated and control group. Values were considered statistically significant when *P* < 0.05, and data was representative of at least three independent experiments.

## 3. Results

### 3.1. LC-QTOF-MS Analysis of OIE

**Figure 1:**
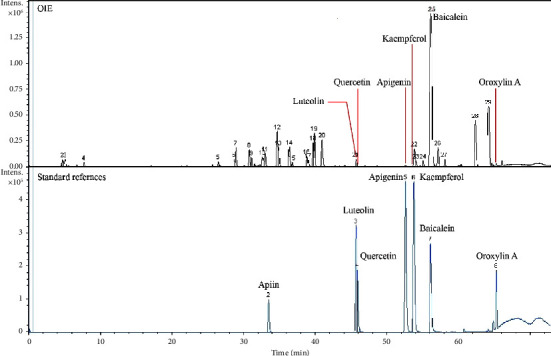
LC-QTOF-MS chromatograms of OIE and standard references.

The peak chromatograms of OIE and reference standards are presented in Figure 1, while the quantitative results are presented in Table 1. Among the seven reference standards used, it was clear that the six major identified flavonoid compounds were luteolin, quercetin, apigenin, kaempferol, baicalein, and oroxylin A. The highest of these compounds was baicalein, and it was found that an OIE concentration of 94.22 *μ*g/mL consisted of baicalein at 3.75 *μ*g/mL.

### 3.2. Cytotoxic Effect of OIE against MDA-MB-231 Cells

**Figure 2:**
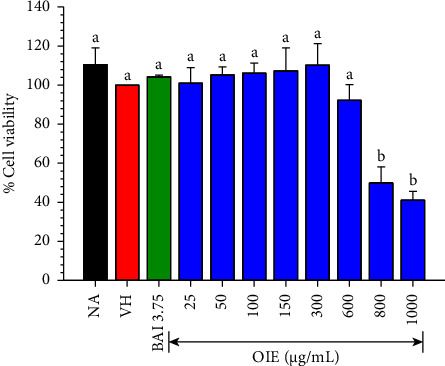
MDA-MB-231 was treated with the indicated concentrations of OIE for 24 hrs, and then cell viability was measured using the CCK-8 assay. Values are expressed as a percentage of the control. The data represent the mean ± SD of three independent experiments and analyses. Bars marked with different letters are significantly different at *P* < 0.05, as determined by one-way ANOVA with the Tukey post hoc test. NA is Naïve, cells alone in media; VH is vehicle control; cells in 0.1% *v*/*v* of DMSO diluted in media; and BAI 3.75 is baicalein 3.75 *μ*g/mL; cells were treated with baicalein 3.75 *μ*g/mL.

MDA-MB-231 cells exhibited different susceptibilities to OIE in a dose-dependent manner (Figure 2). OIE up to a 600 *μ*g/mL concentration had no toxicity towards MDA-MB-231 cells (*P* > 0.05). However, 600 *μ*g/mL of OIE displayed a slightly decreased viability of MDA-MB-231 by 9.5% but insignificant (*P* > 0.05). Therefore, OIE at 25, 50, 100, and 150 *μ*g/mL concentrations should be considered relatively safe for further investigation.

### 3.3. OIE Inhibited the Colony-Forming Ability of MDA-MB-231 Cells

**Figure 3:**
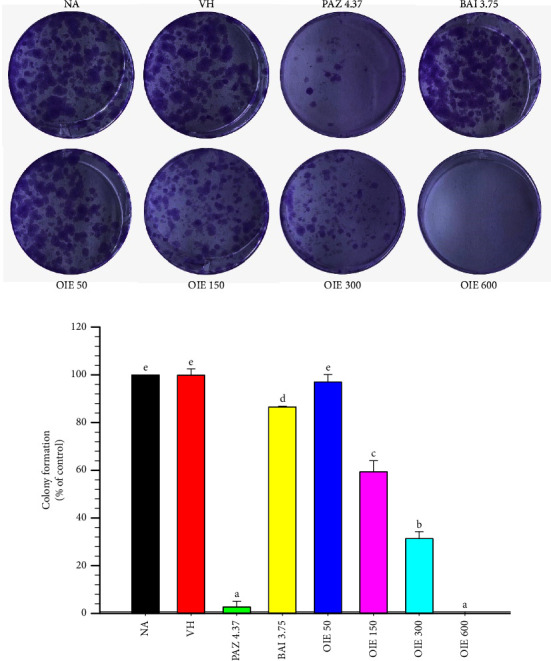
Effect of OIE on colony formation. (a) The representative image of the colony formation after being stained with crystal violet. (b) The number of colonies was counted and expressed as the percentage of control. The data represent the mean ± SD of three independent experiments. NA = Naïve, cells alone in media; VH = vehicle control; cells in 0.1% *v*/*v* of DMSO diluted in media; PAZ 4.37, BAI 3.75, and OIE 50–600 = cells were treated with pazopanib 4.37 *μ*g/mL, baicalein 3.75 *μ*g/mL, and *O. indicum* extract at ranges of 50–600 *μ*g/mL, respectively. Bars marked with different letters are significantly different at *P* < 0.05, as determined by one-way ANOVA with the Tukey post hoc test.

The results showed that OIE treatment significantly inhibited the colony-forming ability of MDA-MB-231 cells starting from 150 to 600 *μ*g/mL compared to the control (*P* < 0.05; Figures 3(a) and 3(b)). The result showed that baicalein decreased the MDA-MB-231 colony formation by approximately 10% (Figure 3(b)). Interestingly, OIE at a concentration of 600 *μ*g/mL was virtually potent as the anticancer drug, pazopanib, in inhibiting MDA-MB-231 colony formation.

### 3.4. OIE Suppressed the Migration of MDA-MB-231 Cells

**Figure 4:**
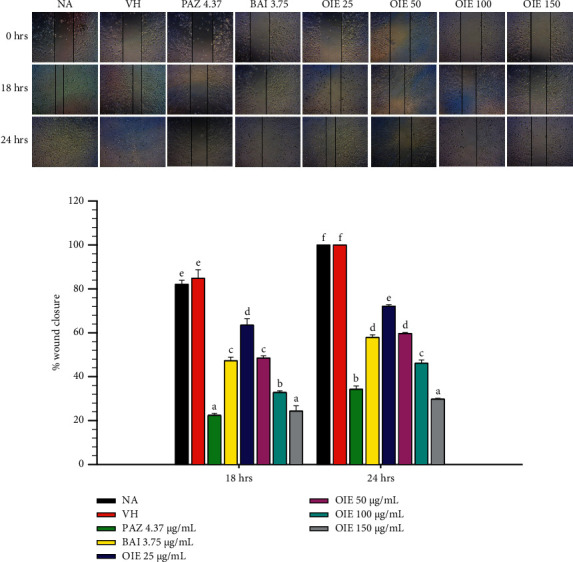
Effects of OIE on the migration of MDA-MB-231 cells. (a) Representative images of wound scratches were taken at 0, 18, and 24 hrs under the inverted microscope (40× magnification). The scale bar in the figure is 100 *μ*m. (b) The migration rate of the cells toward the wounds was expressed as the percentage of wound closure at 18 and 24 hrs. The percentage of wound closure was normalized to the wound area at 0 hrs. The data represent the mean ± SD of three independent experiments. Bars marked with different letters are significantly different at *P* < 0.05, as determined by one-way ANOVA with the Tukey post hoc test. NA = Naïve, cells alone in media; VH = vehicle control, cells in 0.1% *v*/*v* of DMSO diluted in media; PAZ 4.37, BAI 3.75, and OIE 25–150 = cells were treated with pazopanib 4.37 *μ*g/mL, baicalein 3.75 *μ*g/mL, and *O. indicum* extract at ranges of 25–150 *μ*g/mL, respectively.

Treatment with OIE significantly suppressed the closing of wounds in a concentration-dependent manner (Figures 4(a) and 4(b)). The highest concentration of OIE strongly inhibited the migration of MDA-MBA-231 cells, as seen by the decrease in the percentages of wound closure by approximately 25% and 32% after OIE treatment for 18 and 24 hrs, respectively. Interestingly, OIE at 150 *μ*g/mL showed a significantly stronger inhibitory effect on MDA-MB-231 cell migration than baicalein alone (*P* < 0.05) at 18 and 24 hrs of incubation.

**Figure 5:**
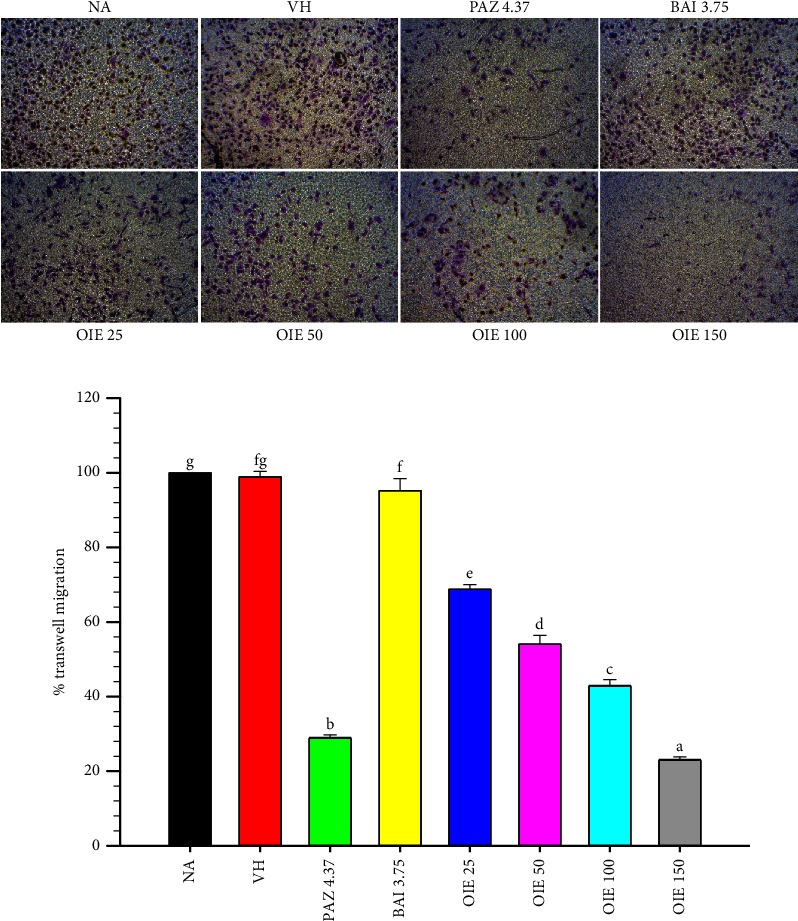
Effect of OIE on MDA-MB-231 cell migration. (a) The images were captured under the inverted microscope (40× magnification). (b) The numbers of invading cells were counted in four different microscopic fields, and the results are expressed as the percentage of migrated cells. The data represent the mean ± SD of three independent experiments with similar results. Bars marked with different letters are significantly different at *P* < 0.05, as determined by one-way ANOVA with the Tukey post hoc test. NA = Naïve, cells alone in media; VH = vehicle control, cells in 0.1% *v*/*v* of DMSO diluted in media; PAZ 4.37, BAI 3.75, and OIE 25–150 = cells were treated with pazopanib 4.37 *μ*g/mL, baicalein 3.75 *μ*g/mL, and *O. indicum* extract at ranges of 25–150 *μ*g/mL, respectively.

The cell migration was further confirmed by the Transwell assay. Similar to the wound healing assay results, OIE significantly decreased MDA-MB-231 cell migration toward a serum chemoattractant in a concentration-dependent manner (*P* < 0.05; Figures 5(a) and 5(b)). As expected, Pazopanib showed a highly antimigration effect on MDA-MB-231 cells, as investigated by wound healing and Transwell migration assay.

### 3.5. Toxicity of OIE on the Chick Chorioallantoic Membrane (CAM) Model

**Figure 6:**
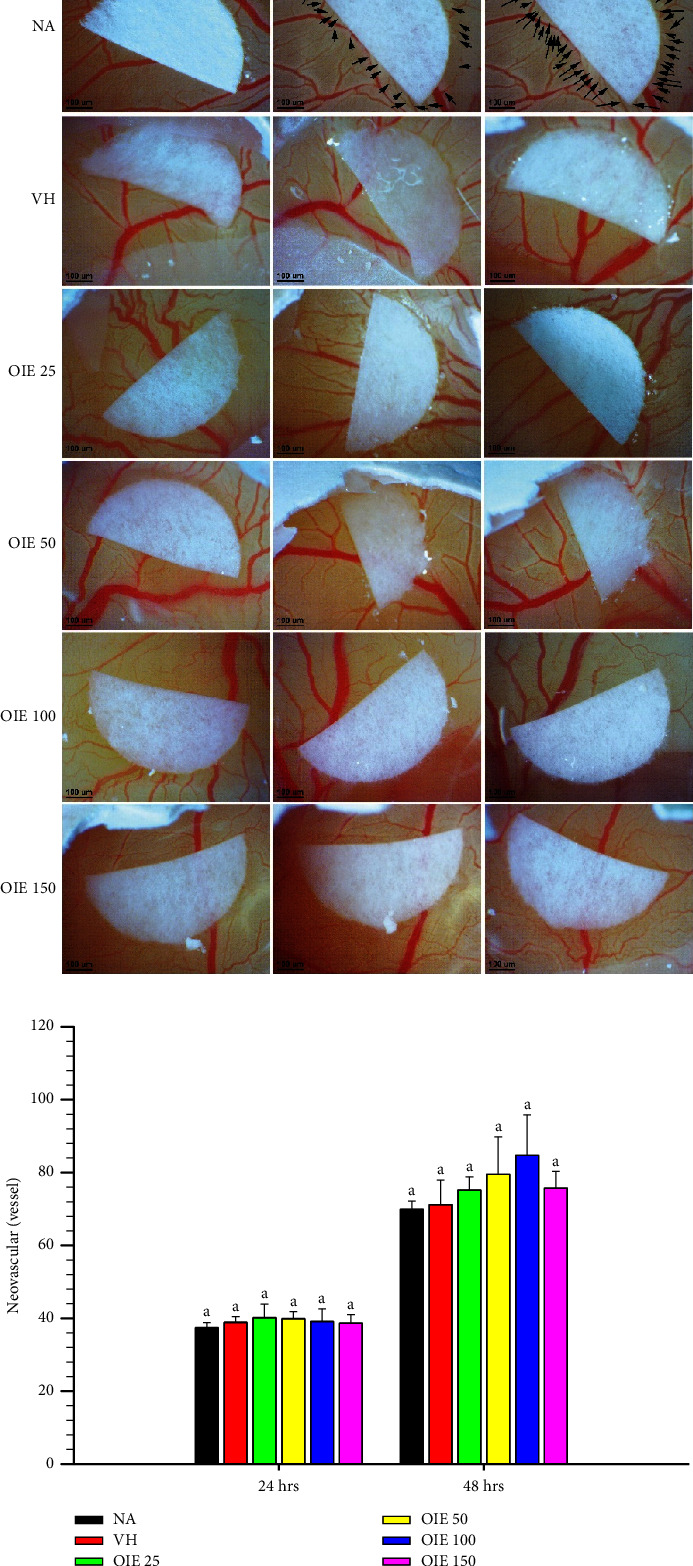
Effect of OIE on normal angiogenesis in the CAM model at 24 and 48 hrs. Values are expressed as means ± SD (*n* = 4) and represent three independent experiments with similar results. Bars marked with different letters are significantly different at *P* < 0.05, as determined by one-way ANOVA with the Tukey post hoc test. NA = Naïve, culture medium; VH = vehicle control, 0.1% *v*/*v* of DMSO diluted in media; OIE 25–150 = cells were treated with *O. indicum* extract at ranges of 25–150 *μ*g/mL, respectively.

The effects of OIE on natural angiogenesis in the CAM was evaluated at 24 and 48 hrs of postexposure, as shown in Figures 6(a) and 6(b). The results demonstrated that the exposure to OIE ranging from 25 to 150 *μ*g/mL for 24 and 48 hrs was not significantly different in the number of neovascular formations compared to VH (*P* > 0.05). Therefore, a concentration of up to 150 *μ*g/mL of OIE was chosen for subsequent antiangiogenesis study.

### 3.6. Antiangiogenic Activity of OIE on MDA-MB-231-Induced Angiogenesis in the CAM Model

**Figure 7:**
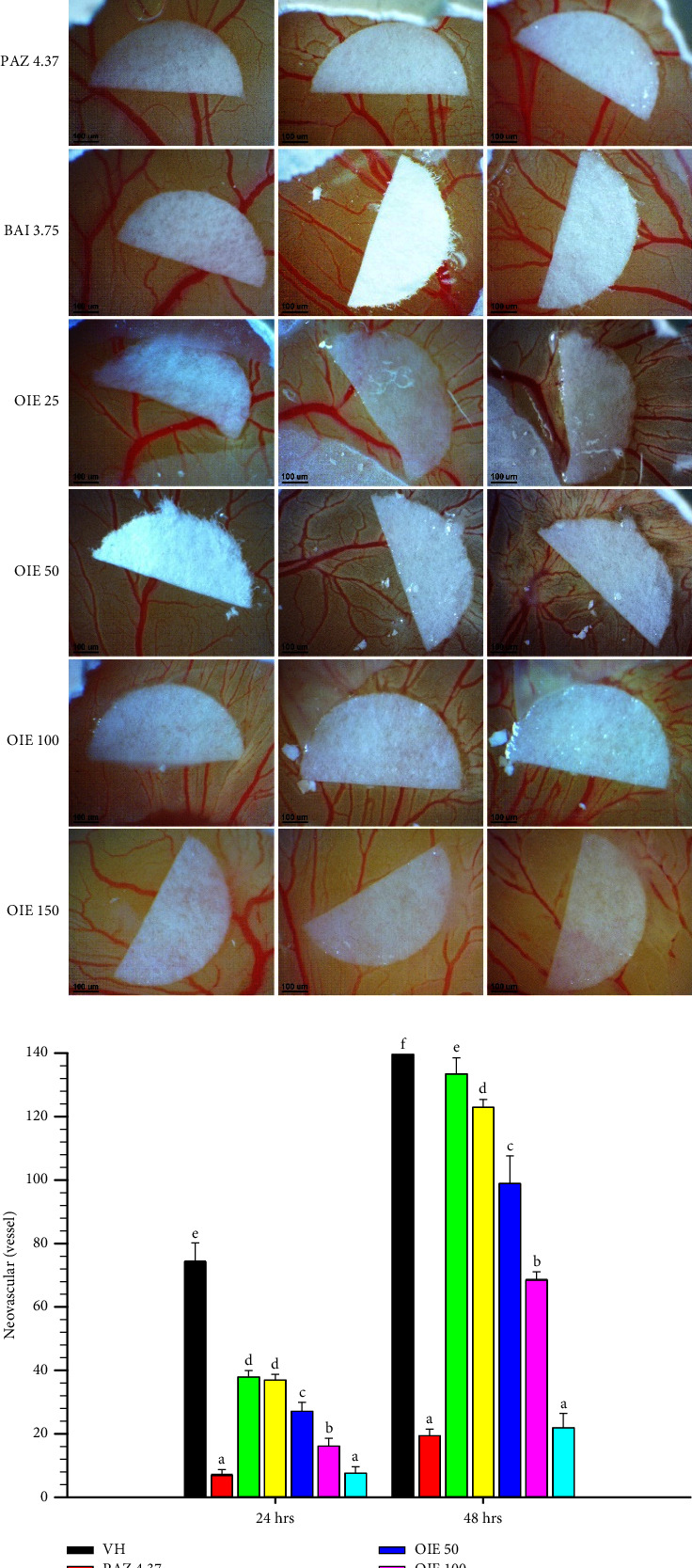
Antiangiogenic activity of OIE on MDA-MB-231-induced angiogenesis in the CAM model at 24 and 48 hrs. Values are expressed as means ± SD (*n* = 4) and represent three independent experiments with similar results. Bars marked with different letters are significantly different at *P* < 0.05, as determined by one-way ANOVA. NA = Naïve, cells alone in media; VH = vehicle control, cells in 0.1% *v*/*v* of DMSO diluted in media; PAZ 4.37, BAI 3.75, and OIE 25–150 = cells were treated with pazopanib 4.37 *μ*g/mL, baicalein 3.75 *μ*g/mL, and *O. indicum* extract at ranges of 25–150 *μ*g/mL, respectively.

OIE clearly produced a dose-dependent suppression of MDA-MB-231-induced neovascularization in the CAM model at both 24 and 48 hrs (*P* < 0.05; Figures 7(a) and 7(b), respectively). The highest concentration of OIE at 150 *μ*g/mL caused the reduction of MDA-MB-231-induced neovascularization by 89% and 86% at 24 and 48 hrs, respectively, compared to VH control (MDA-MB-231-induced neovascularization in CAM). Likewise, the number of neovascularization induced by MDA-MB-231 was also reduced by approximately 49% and 20% after exposure to baicalein at 24 and 48 hrs, respectively. As expected, pazopanib exhibited a strong antiangiogenic potential by reducing MDA-MB-231-induced neovascularization by approximately 88–90% compared to VH control at 24 and 48 hrs.

## 4. Discussion

Breast cancer is the second-most common life-threatening disease in women worldwide [26]. The two main reasons for the high mortality rates associated with breast cancer and the leading causes of poor clinical outcomes are invasion and metastasis [27]. This study was particularly interested in using highly metastatic, aggressive breast cancer, MDA-MB-231 cell lines, as a model for investigating the anticancer activity of OIE.

Before investigating anticancer activity, we tested the cytotoxicity of OIE against MDA-MB-231 cell lines. In a subsequent experiment, the noncytotoxic concentration of OIE up to 600 *μ*g/mL was chosen to treat MDA-MB-231. Cancer is any one of many diseases characterized by the development of abnormal cells that divide uncontrollably [3]. The ability to self-renew and differentiate gives rise to the heterogeneous phenotype of the tumor cells. These cells are believed to be involved in cancer metastasis, recurrence, and therapy resistance [28]. The colony formation assay was applied to detect the ability of single cells to survive and reproduce to form colonies after OIE treatment. This result revealed that OIE demonstrated a long-term suppressive effect on cell proliferation of MDA-MB-231 cells in a concentration-dependent manner.

Cell migration and invasion play an essential role in the development of cancer. Regardless of advancements in local cancer treatments, there is a higher chance of clinical challenges in combating systemic metastatic disease [29]. The wound healing assay is straightforward and gives valuable initial information about cell front migration but does not provide dynamic information. The transwell migration was further used to analyze the ability of a single cell to respond to chemoattractant directionally. The wound healing and transwell migration experiments were performed on MDA-MB-231 breast cancer cells to investigate the antimigration activity of OIE. To compromise on excluding the cytotoxic MDA-MB-231, a concentration of up to 150 *μ*g/mL of OIE was selected for investigation in the experiment. The wound healing assays showed that OIE significantly suppressed cell migration of MDA-MB-231 breast cancer cells in a dose-dependent manner (Figures 4(a) and 4(b)). The migratory function of cells was confirmed by transwell migration, showing that OIE drastically inhibited the migration of MDA-MB-231 breast cancer cells. Together, these results provide evidence that OIE could hinder metastasis progression in breast cancer. Results similar to this have also been reported by Kumar et al. [18], who found that the petroleum ether hot extract of *O. indicum* demonstrated an apparent inhibition of cell migration and proliferation in MDA-MB-231 cells. This is consistent with earlier findings that *O. indicum* leaf and fruit extracts exhibit an anticancer effect on MCF-7 breast cancer cells by inhibiting colony formation and cell migration, reducing MMP-9 and ICAMP1 gene expression, and MMP-9 protein expression [19, 20]. Expressions of MMP-9 and ICAMP1 are closely linked to the growth, invasion, metastasis, and angiogenesis of cancer cells [30–32]. This literature triggers us to believe that *O. indicum* may also be able to reduce angiogenesis. Therefore, the antiangiogenic effects of OIE were further investigated using the CAM model.

Angiogenesis is a term that describes the formation of new blood and lymphatic vessels from pre-existing vasculature. Angiogenesis plays a crucial role in tumor growth, invasion, and metastasis of cancer diseases; therefore, inhibiting angiogenesis could be a strategy to arrest tumor growth and metastasis [33, 34]. The present study elucidated for the first time the antiangiogenesis properties of *O. indicum* toward breast cancer cells-induced angiogenesis. The antiangiogenesis study revealed that OIE showed a full antiangiogenesis effect without any substantial toxic effect on the cells. These results substantially agree with the results of Talari et al. [21], showing that *O. indicum* exhibits the antiangiogenic effect on VEGF-induced neovascularization in the CAM model. In addition, this study also elucidates for the first time the antiangiogenesis effect of *O. indicum* on breast cancer cell-induced angiogenesis in the CAM model.

In this study, the analysis of phytochemical constituents using the LC-QTOF-MS instrument attested to the presence of baicalein (39.75 *μ*g/mg of OIE), with a high amount in OIE. It was found that an OIE concentration of 94.22 *μ*g/mL consisted of baicalein at 3.75 *μ*g/mL. These results correspond with the previous study of Dunkhunthod et al. [8, 11] that the amount of baicalein contains in OIE with a concentration range of 25.50–32.85 *μ*g/mg of the extract. To clarify whether baicalein contributed to the anticancer activity of OIE or not. So baicalein was used as a positive control. Many studies have shown that baicalein exhibits anticancer properties, which are attributed at least partially to the inhibition of cell proliferation, cell migration, and invasion in many cell types, including AGS human gastric cancer cells, DLD1 colorectal cancer cells, B16F10 melanoma cells, vein endothelial cells (HUVECs), and MDA-MB-231 breast cancer cells [28, 35–37]. Likewise, many researchers have also reported the antiangiogenic activity of baicalein [38–40]. Similar to other investigators, this study further confirmed the anticancer activity of baicalein since baicalein showed the inhibition of colony formation, cell migration, and angiogenesis in breast cancer MDA-MB-231 cells. Compared to OIE, baicalein (3.75 *μ*g/mL) alone showed a lower anticancer potency than OIE at 100 and 150 *μ*g/mL concentrations throughout each investigation. The higher strength of OIE than baicalein may be due to OIE's other bioactive compounds, such as luteolin, apigenin, quercetin, and other compounds. These compounds have been demonstrated to possess anticancer properties and suppress metastasis and angiogenesis progression in several cancers [41–44]. These reports lend support to the assumption that synergistic activity may occur between baicalein and other flavonoid compounds in enhancing the anticancer activity of OIE.

In the present study, the anticancer mechanism of OIE was compared by using pazopanib as a positive anticancer drug. Pazopanib is a pan-vascular endothelial growth factor receptor inhibitor, and preclinical work indicates that pazopanib exerts an anticancer effect by inhibiting both angiogenic and oncogenic signalling pathways [45, 46]. As expected, pazopanib exhibits a strong anticancer potency by suppressing colony formation, cell migration, and angiogenesis in breast cancer MDA-MB-231 cells. Interestingly, the OIE at 150 *μ*g/mL was as practically efficient as the positive anticancer drug, pazopanib, at 4.37 *μ*g/mL, in inhibiting MDA-MB-231 cell migration and angiogenesis induced by these cells.

## 5. Conclusions

The findings in the current study lead us to believe that OIE possesses anticancer activity by suppressing colony formation, cell migration, and MDA-MB-231-induced angiogenesis. The present comprehensive study provides evidence that baicalein is also responsible for OIE's anticancer activity. The underlying mechanism behind the antimigratory and antiangiogenic effects of OIE in MDA-MB-231 breast cancer cells should be further investigated. Nevertheless, this inhibition of cell proliferation, cell migration, and tumor-induced angiogenic activities in OIE could explain, at least in part, the claimed anticancer activity of *O. indicum*.

## Figures and Tables

**Table 1:** LC-QTOF-MS data of phytochemical compounds in OIE.

Compound names	EIC	Rt (min)	Quantification (*μ*g/mg of OIE)
Apiin	563	33.5	Not detected
Luteolin	285	45.7	0.158
Quercetin	301	45.9	0.044
Apigenin	269	52.6	0.028
Kaempferol	285	53.7	0.004
Baicalein	269	56.1	39.750
Oroxylin A	283	65.3	0.238

## Data Availability

The datasets used and analyzed during this study are available from the corresponding author upon reasonable request.
